# Mumps Orchitis: Clinical Aspects and Mechanisms

**DOI:** 10.3389/fimmu.2021.582946

**Published:** 2021-03-18

**Authors:** Han Wu, Fei Wang, Dongdong Tang, Daishu Han

**Affiliations:** ^1^ Department of Immunology, Shenzhen University School of Medicine, Shenzhen, China; ^2^ Institute of Basic Medical Sciences, School of Basic Medicine, Peking Union Medical College, Chinese Academy of Medical Sciences, Beijing, China; ^3^ Reproductive Medicine Center, Department of Obstetrics and Gynecology, The First Affiliated Hospital of Anhui Medical University, Hefei, China

**Keywords:** mumps, MuV, orchitis, testis, infertility

## Abstract

The causative agent of mumps is a single-stranded, non-segmented, negative sense RNA virus belonging to the *Paramyxoviridae* family. Besides the classic symptom of painfully swollen parotid salivary glands (parotitis) in mumps virus (MuV)-infected men, orchitis is the most common form of extra-salivary gland inflammation. Mumps orchitis frequently occurs in young adult men, and leads to pain and swelling of the testis. The administration of MuV vaccines in children has been proven highly effective in reducing the incidence of mumps. However, a recent global outbreak of mumps and the high rate of orchitis have recently been considered as threats to male fertility. The pathogenesis of mumps orchitis remains largely unclear due to lack of systematic clinical data analysis and animal models studies. The alarming increase in the incidence of mumps orchitis and the high risk of the male fertility have thus become a major health concern. Recent studies have revealed the mechanisms by which MuV-host cells interact and MuV infection induces inflammatory responses in testicular cells. In this mini-review, we highlight advances in our knowledge of the clinical aspects and possible mechanisms of mumps orchitis.

## Introduction

Mumps is a worldwide contagious disease caused by mumsp virus (MuV). MuV is mainly transmitted via the respiratory route. MuV infection results in painful inflammatory symptoms, such as parotitis, orchitis, oophoritis, aseptic meningitis, encephalitis and pancreatitis (1). Besides the typical painfully swollen parotitis in infected males, orchitis is the most common extra-salivary inflammation and an important etiological factor of male infertility (2).

Mumps orchitis generally manifests around a week after the onset of parotitis (1, 3). Approximately 30% of mumps orchitis in post pubertal males suffer from infertility or subfertility (3). Mumps orchitis may lead to the atrophy of germinal epithelium with spermatogenesis arrest and the disruption of steroidogenesis. MuV complications are not lethal, therefore lacking human samples to examine disease pathogenesis.

Current information on pathogenesis after MuV infection is largely based on investigation using animal models (4–8). We have recently examined mechanisms underlying MuV infection of testicular cells, MuV-induced immune responses and impairment of testicular functions in mouse models, which provide novel insights into the pathology of mumps orchitis and related male infertility (9–11). In this mini review, we briefly summarize MuV biology and focus on mechanisms underlying pathogenesis of mumps orchitis.

## MuV and Complications

**Figure 1:**
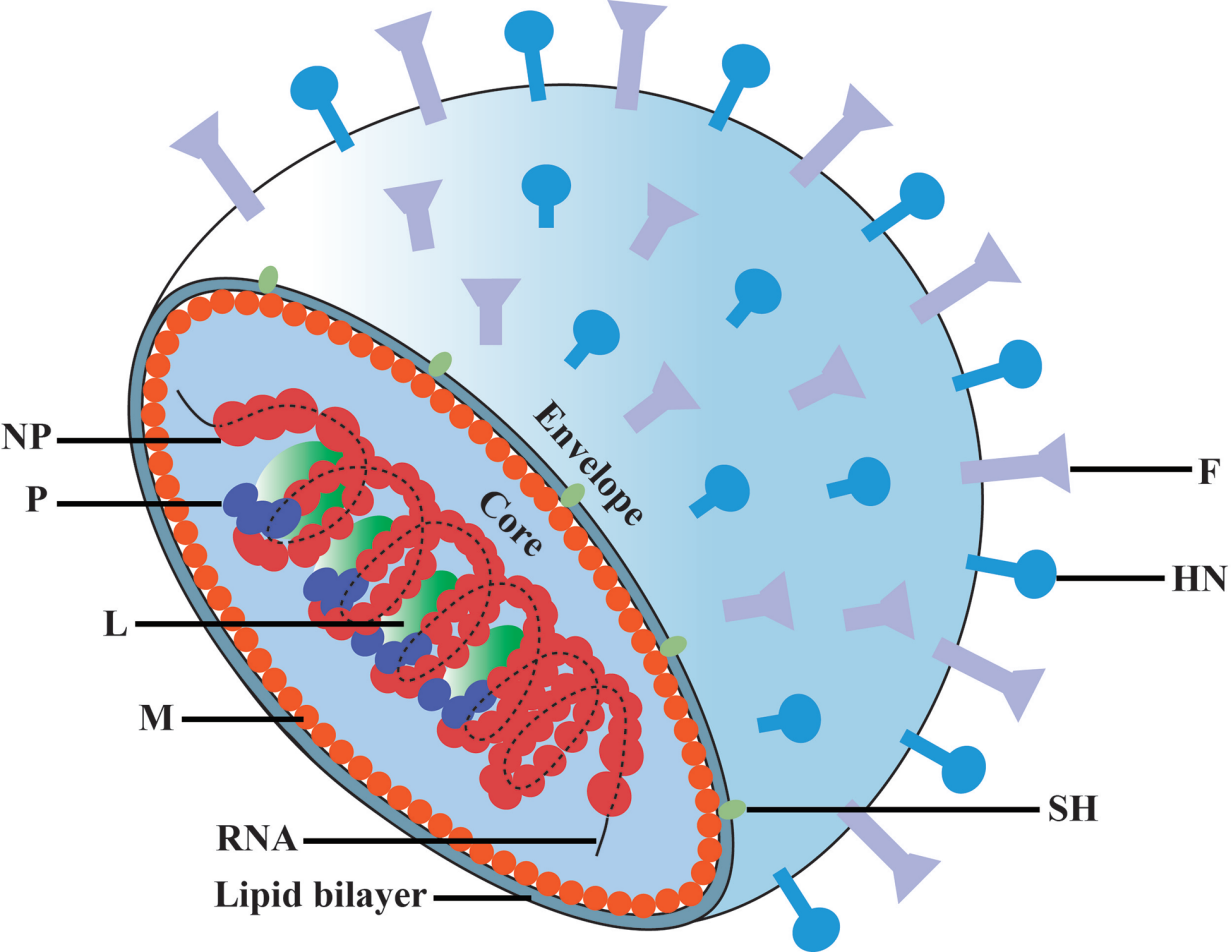
Schematic diagram of mumps virus. The MuV core is enveloped by matrix protein (M) and lipid bilayer containing small hydrophobic protein (SH), spike fusion protein (F) and hemagglutinin-neuramindase (HN). The genomic RNA combines with nucleoprotein (NP), polymerase (L) and phosphoprotein (P) to form the core of the helical nucleocapsid.

MuV belongs to the *Paramyxoviridae* family, which consists of enveloped particles that contain a non-segmented single negative-strand RNA genome (**Figure 1**). The MuV genome consists 15,384 nucleotides that encodes seven proteins: nucleoprotein (NP), polymerase (L), phosphoprotein (P), matrix protein (M), hemagglutinin/neuraminidase (HN), fusion protein (F), and small hydrophobic protein (SH) (12). MuV strains can be classified into 12 genotypes based on the nucleotide diversity of the SH gene (13, 14). Viral RNA encapsulated by NP is the template for replication. A complex of L and P acts as a replicase to transform the negative-strand RNA to a positive-strand RNA and as a transcriptase to generate mRNA. HN and F glycoproteins cooperatively mediate virus attachment and internalization to host cells via its receptor sialic acid that is present on the surface of most mammalian cell types.

MuV is initially transmitted via the respiratory route by the inhalation of contaminated droplets from an infected respiratory tract. Based on the array of symptoms, MuV would initially replicate within the lymphoid and reticuloendothelial tissue of the respiratory tract, which then lead to a transient viremia that may spread viruses into multiple organs (12). While it is assumed that MuV first infects the respiratory epithelium, the primary target cells for early infection and replication remain unclear. However, MuV has rarely been detected in blood, probably due to the coincident development of specific antibodies.

Besides asymptomatic and mild respiratory diseases in approximately 30% of infections, the typical characteristics of mumps include swollen parotitis, which is used to diagnose the disease. Parotid gland swelling is mostly bilateral, occurs 2−3 weeks after transmission, and lasts for 2−3 days. The symptom may persist for a week or more in minor cases. Following parotitis, MuV infection can lead to inflammation in the reproductive and central nerve systems, including orchitis, oophoritis, encephalitis, and meningitis (1). MuV may also result in myocarditis, pancreatitis and nephritis. While the complications are mostly self-limiting, long-term sequelae such as infertility, paralysis, hydrocephalus and deafness can occur. Mumps orchitis is the most concerned extra-parotid gland inflammation due to its detrimental effect on human reproduction.

## Clinical Aspects of Mumps Orchitis

### Vaccination and Incidences of Mumps Orchitis

**Figure 2:**
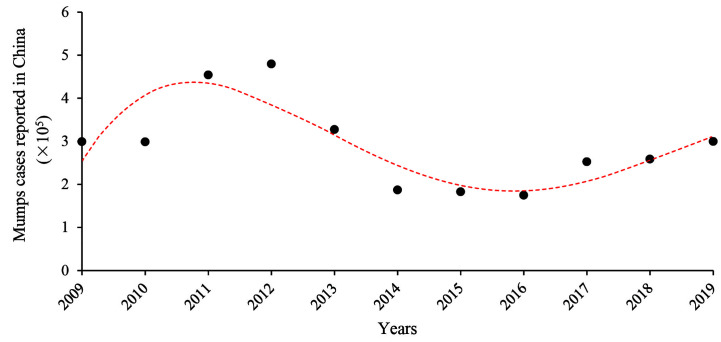
Annual mumps cases reported in China from 2009 to 2019. Data come from reports of the National Health Commission of China.

In the pre-vaccine era, mumps was an infectious disease that was most commonly transmitted among children and was mainly complicated with parotitis (15, 16). With routine MuV vaccination, mumps incidence has dramatically declined. However, there have been large outbreaks of mumps worldwide in the past decades, including vaccinated populations (17–31). In China, the mumps vaccine was first introduced in the National Immunization Program in 2007 (32). From June 2020, the policy had changed to receive two doses of trivalent** **measles, mumps and rubella (MMR) vaccine. The annual occurrence of mumps cases from 2009 to 2019 is shown in **Figure 2**. After high vaccination coverage, mumps incidence dropped by half in 2016 from its peak in 2012. Notably, mumps incidences slightly increased from 2017 to 2019. This may be due to the fact that more than 10 years have passed since the first one dose vaccine was administered, and vaccine effectiveness has declined.

The recent resurgence of mumps mainly involved adolescents and young adults, with high rates of orchitis frequently reported (3, 33–35). Orchitis is the most common complication of mumps and occurs in as high as 40% of all mumps cases in young adult men (36). Mumps orchitis is mostly unilateral, but can occur bilaterally in 10−30% of cases (37). Unvaccinated postpubertal males are susceptible to virus outbreaks and are at high risk of developing mumps orchitis. In China, the vaccination rate of men born in the 1990s, who are now 30 years old, is low due to lacking MMR vaccination program in less developed areas. Thus, it is essential to be aware of this epidemiological shift and the resurgence of mumps orchitis in the clinic. Cases of orchitis following MMR vaccination are reported in post-pubertal adults, suggesting that the MMR vaccination may have an adverse effect on the testis in certain young adults (38–41). Therefore, whether unvaccinated male in this age group should be offered the MMR vaccine requires in-depth and carefully evaluation.

### Clinical Manifestations

Mumps orchitis usually occurs at about one week after the onset of parotitis in young adult males with MuV infection. The onset of orchitis is associated with constitutional symptoms, such as headache and fever and later manifests as testicular swollen and pain. Examination of the scrotum generally indicates swelling testes, associated tenderness, and inflammation of the scrotum. Epididymitis also occurs in most of the mumps orchitis cases and results in mumps epididymo-orchitis (42, 43). A recent study demonstrates that the epididymal head is mostly involved in mumps epididymo-orchitis, which is in contrast to bacterial epididymitis that commonly occurs in the cauda epididymis (44, 45). During the acute phase, the endocrine function of the testes is altered, e.g., decreased testosterone levels. Some cases also show increased luteinizing hormone (LH) and follicle-stimulating hormone (FSH) levels (46). The acute symptoms can resolve within two weeks; however, testicular atrophy can occur in half of the orchitis patients and is characterized by an oblong shape, low echogenicity, and decreased vascularity based on ultrasonographic findings (3, 47). However, seminal abnormalities, including sperm count, motility and morphology, may sustain for years after recovery (37), suggesting the abnormal spermatogenesis can occur.

### Diagnostic and Therapeutic Approaches

There is no standard criteria procedure for MuV diagnosis because it is not a common condition that is observed in hospital. Diagnosis of MuV is mainly based on clinical complication and laboratory testing. Orchitis characteristically presents with swollen and pain testes. Ultrasonography can provide image features, including low echogenicity, hypervascularity, and increased volume of the testes and epididymis (43, 48). Testicular inflammation and hydrocele could also be detected. The routine urine analysis is necessary for diagnosing the mumps orchitis to rule out bacterial infection (33).

The definitive diagnosis of mumps orchitis should be based on laboratory tests. The presence of MuV in saliva or seminal fluid can be determined by real time RT-PCR. The MuV-specific IgM and IgG antibodies in blood can be measured by ELISA for serological markers of MuV infection. A positive serum IgM or a four-fold increase in IgG level is considered serologically positive for MuV infection (49). While MuV can be isolated from the seminal fluid within two weeks after symptom onset (50), the test for viral infectivity is usually not performed in the clinical diagnosis due to the complicated procedure for this test.

MuV infection is mostly self-limiting, and there is currently no specific antiviral therapy available. The treatment for mumps orchitis generally includes supportive procedures, including bed rest, scrotal support, and analgesic and anti-inflammatory drugs against pain and fever. Symptoms can resolve with treatment in 4−10 days (51). Steroid drugs were used to diminish testicular pain and swelling, but it does not alter the clinical course and prevent subsequent atrophy. Interferon has been used in a series of cases to cure mumps orchitis; however, this is a controversial method because there is conflicting evidence on its therapeutic effect. Erpenbach et al. claimed to have prevented testicular damage and infertility in four patients who had bilateral mumps orchitis by using systematic interferon for seven days (52). However, Yeniyol et al. found that interferon is not effective in preventing testicular atrophy because 40% of patients presents evidence of total atrophy of seminiferous tubules on testicular biopsies performed during follow-up (53). A recent study that assessed a series of 56 cases of mumps orchitis treated with interferon also showed some kind of hormone or sperm impairment in most patients during the later follow up period, while only two patients (14%) were considered free of sequelae (3). Although some treatments can diminish the complications of acute mumps orchitis, the preventive and therapeutic approaches of the orchitis-caused testicular damage and subfertility/infertility require establishment.

## Mechanisms of Mumps Orchitis

**Figure 3:**
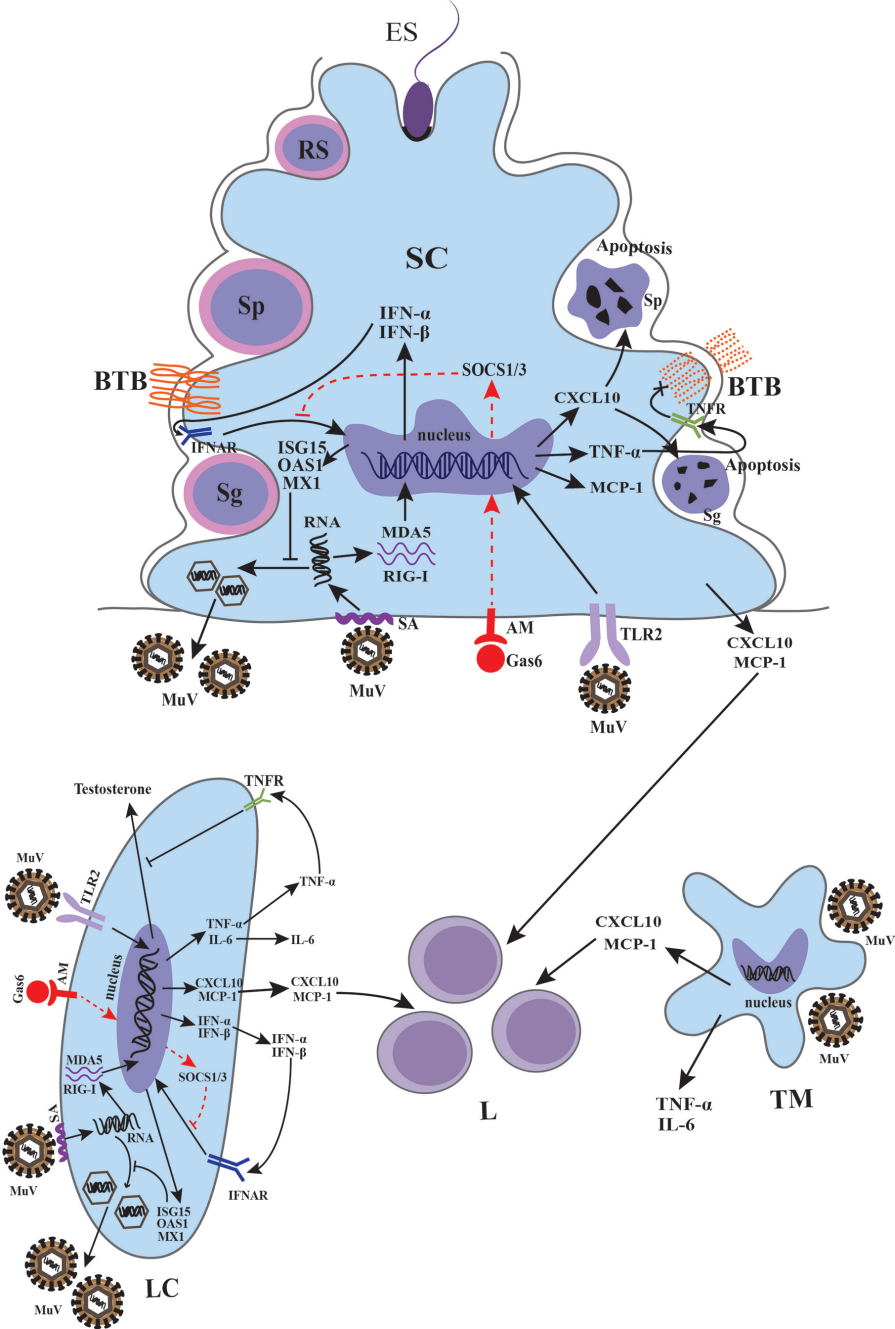
Schematic of MuV infection of testicular somatic cells and downstream effects. Sialic acid (SA) on the surface of Sertoli cells (SCs) and Leydig cells (LCs) mediated MuV entry into cells. Gas6 and Axl/Mer (AM) receptor tyrosine kinase system facilitates MuV replication by inhibiting antiviral response. MuV triggers Toll-like receptor 2 (TLR2) and cytosolic RNA sensors MDA5/RIG-I signaling pathways, thereby inducing the expression of various immunoregulatory cytokines, including pro-inflammatory factors TNF-α and IL-6, chemokines CXCL10 and MCP-1, and type 1 interferons INF-α and IFN-β. IFN-α and IFN-β then induce the expression of various proteins, including ISG15, OAS1 and Mx1, that can inhibit MuV replication. MuV infection also induces the production of CXCL10, MCP-1, TNF-α and IL-6 by testicular macrophages (TM). CXCL10 produced by SC in response to MuV infection induces apoptosis of germ cells, whereas TNF-α disrupts blood-testis barrier (BTB) integrity and permeability. MuV infection of LC inhibits testosterone synthesis. MuV-induced TNF-α is presumably responsible for the MuV inhibition of testosterone synthesis. MCP-1 and CXCL10 produced by SC, LC and TM may recruit leukocytes (L), resulting in orchitis. Sg, spermatogonium; Sp, spermatocyte; RS, round spermatid; ES, elongated spermatid. SOCS, suppressor of cytokine signaling. →, promotion; ┴, inhibition; ×, disruption of BTB. The red dashed line indicates a possible signaling pathway in SCs and LCs according to previous findings (54–56).

It is difficult to study the pathogenesis of mumps orchitis in humans due to the lack of samples. Recent studies using mouse models provide insights to mechanisms by which MuV infects testicular cells and impairs testicular functions (**Figure 3**).

### MuV Receptors and Testis Tropism

It is well-known that MuV has high tropism to the testis (57). Thus, understanding the mechanism of receptor recognition by MuV is very important. Sialic acid, which is expressed on the cell surface as a terminal component of sugar chains, plays a role in mediating infection of many viruses, including influenza viruses (58), the Middle East respiratory syndrome coronavirus (59), and Zika virus (ZIKV) (60). A previous study demonstrated that MuV used a trisaccharide containing α2,3-linked sialic acid on the cell surface as a receptor that interacts with MuV attachment protein HN (61). The MuV-HN-receptor interaction triggers the activation of the F protein, causing fusion of the viral envelope with the plasma membrane and allowing cell entry (62). A very recent study has confirmed the presence of sialic acid on the surface of Sertoli cells (SCs) and Leydig cells (LCs) (63). Depletion of sialic acid by sialidase decreases MuV internalization into SCs and LCs, but does not affect MuV binding to these cells (63). These results suggest that other co-receptors for MuV binding to testicular cells exist. Recently, two novel glycan motifs, sialyl Lewis^X^ (SLe^X^) and the GM2 ganglioside (GM2-glycan), have been shown to serve as MuV receptors (64). Whether these receptors function for MuV tropism toward testicular cells requires further investigation.

Other types of cell receptors such as AXL and MER, which are members of a subfamily of receptor tyrosine kinases, have been suggested as potential candidates for MuV tropism. AXL and MER are abundantly expressed in SCs and LCs and play important roles in regulating testicular immune privilege (65). Both AXL and MER can interact with the ligands Gas6 and protein S that in turn bind to the surface of enveloped viruses (66). Several studies have demonstrated that AXL facilitates infection of multiple viruses, including Ebola virus (67), influenza virus (68), and ZIKV (69). AXL and MER have been suggested to function as binding or entry factors for MuV based on the observation that these are highly expressed in the testis during MuV infection (63, 70). Knockout of *Axl*, *Mer*, and both *Axl* and *Mer* did not affect MuV binding to SCs and LCs, and the levels of MuV internalized into SCs and LCs were comparable (63). Thus, AXL and MER were not required as binding or entry factors for MuV in these cells. However, a genetic study showed that double knockout of both *Axl* and *Mer* remarkably decreased MuV replication, whereas single knockout of either *Axl* or *Mer* barely affected MuV replication. Additionally, a study using an inhibitor of AXL and MER showed no change in MuV replication in type 1 interferon receptor knockout (*Ifnar1* ^−/−^) SCs and LCs, while it significantly decreased in wild-type cells, suggesting a redundant role of AXL and MER in facilitating MuV replication in SCs and LCs by inhibiting the antiviral IFN signaling (63). These findings on the mechanism underlying receptors-mediated MuV binding, internalization and replication (**Figure 3**) may be helpful discovering the targets for the prevention of MuV infection.

### MuV-Induced Immune Response in Testicular Cells

Due to the lack of testicular biopsy from mumps orchitis patients, it is difficult to investigate MuV infection and pathogenesis in the testis. Although humans are believed to be the only natural reservoir, MuV was experimentally used to infect various animal models to evaluate protective immunity against MuV (8, 71). Unfortunately, studies on the pathogenesis of orchitis in the animal models are limited. A current study found that MuV can infect the majority of mouse testicular cells, including SCs, LCs, testicular macrophages (TMs), and male germ cells (GCs) (9). However, MuV differentially replicates in these testicular cells. MuV replicated at relatively high efficiency rate in SCs compared with LCs and TMs. In contrast, MuV does not replicate in male GCs. These findings suggest that testicular cells exhibit different innate antiviral responses against MuV replication.

To understand the mechanisms underlying MuV-induced orchitis, a recent study investigated the pattern recognition receptors-initiated innate immune responses of testicular cells to MuV infection (11). MuV induces innate immune responses in mouse SCs and LCs through the activation of Toll-like receptor 2 (TLR2) and retinoic acid-inducible gene I (RIG-I) signaling pathways. MuV-initiated TLR2 signaling mainly induces pro-inflammatory cytokines (TNF-α and IL-6) as well as chemokines (MCP-1 and CXCL10), whereas the RIG-I pathway principally participated in the induction of type 1 interferons (IFN-α and IFN-β). In response to MuV infection, SCs produced relatively high levels of pro-inflammatory cytokines and chemokines, but low levels of IFN-α and IFN-β compared to LCs. A previous study using a mouse model showed that mumps-associated gene geranylgeranyl diphosphate synthase 1 deficiency in SCs resulted in male infertility and abnormal activation of MAPK and NF-κB downstream of TLR2 signaling (72). These investigations suggest that TLR2 plays a crucial role in initiating the innate immune responses to MuV infection in testicular cells. However, the TLR2 ligand in MuV has yet to be identified. In contrast, TNF-α, IL-6, MCP-1, CXCL10, IFN-α and IFN-β were nearly undetectable in male GCs after MuV infection, suggesting a low level of innate immune response in GCs (11). MCP-1 and CXCL10 produced by TMs, SCs and LCs in response to MuV infection should facilitate the recruitment of leukocytes into the testis for MuV orchitis (**Figure 3**).

Usually, viral replication in infected cells is restricted by cellular innate antiviral responses. The production of IFNs is a universal mechanism of the host’s defense against viral infection (73). IFN-β can inhibit MuV replication in SCs, LCs, and TMs by inducing the expression of antiviral proteins, including ISG15, OAS1, and MX1, but not in GCs. Remarkably, GCs and TMs are equipped with autophagy machineries, and autophagy restricts MuV replication in these cells. In contrast, autophagy is not involved in limiting MuV replication in LCs and SCs. These findings suggest a cell type-specific innate antiviral mechanisms against MuV replication in testicular cells.

Notably, viral infection in male GCs may be sexually transmitted to female partners and fetus, thus leading to virus parallel and vertical transmission (74). The antiviral defense of male GCs is particularly important not only for male fertility but also for limiting virus transmission. The innate antiviral responses in most type of cells after viral infection produce type 1 IFNs and various pro-inflammatory cytokines (75). The increased levels of certain pro-inflammatory cytokines can be harmful to spermatogenesis (76). However, autophagy directly uptakes and degrades viruses that invade GCs, without the induction of pro-inflammatory cytokines (77). Therefore, autophagy of male GCs should be suitable for the antiviral defense without harming spermatogenesis.

### MuV Infection Damages Testis Function

The cytokines induced by viral infection can mediate organ dysfunction and tissue damage (78). We recently found that MuV infection induced the production of various pro-inflammatory cytokines and inhibited testosterone synthesis in LCs (11). The production of pro-inflammatory cytokines such as TNF-α by LCs after MuV infection may be responsible for the inhibition of testosterone synthesis by reducing the expression of enzymes for testosterone synthesis (79). However, one study showed that the testicular testosterone level was reduced in TNF-α knockout mice, which was probably caused by the augmentation of Mullerian inhibiting substance in the testis (80). TNF-α may play dual roles in the protection and disruption of the tissue functions dependent on its level under pathophysiological conditions. The high level of TNF-α in the testis due to infection and inflammation disrupts testicular functions (81, 82). Moreover, we demonstrated that the production of TNF-α and CXCL10 in SCs after MuV infection impaired blood-testis barrier (BTB) and induced GC apoptosis, respectively (10, 83). The BTB plays an important role in maintaining normal spermatogenesis. TNF-α produced by SCs in response to MuV infection impaired BTB formation by reducing the levels of occludin and zonula occludens 1 (ZO-1) (83). The finding is supported by a previous study that occludin is one of the target proteins of TNF-α in rat SCs (84). Moreover, a recent study showed that the exposure of SCs to inflammatory mediators derived from ZIKV-infected macrophages also led to the degradation of the ZO-1 protein, which correlated with increased BTB permeability (85). These findings suggest an adverse role of virus-induced production of inflammatory cytokines such as TNF-α. During spermatogenesis, a large number of novel antigens are produced by post-meiotic spermatids in seminiferous tubules after immune self-tolerance has been established (86). The production of autoantibodies against GC antigens is a common feature for orchitis patients. This may explain why mumps orchitis often causes male infertility in postpubertal and young adult men but rarely affects children when the spermatids have not yet been produced in the testes.

The deleterious effects of MuV infection on male GCs have also been examined in a recent study (10). The study showed that TNF-α produced by mouse SCs in response to MuV induced CXCL10 expression in autocrine manner. CXCL10 is a pleiotropic cytokine capable of exerting various functions, including the chemotaxis of leucocytes and induction of apoptosis (87). CXCR3, as the receptor of CXCL10, is expressed on the surface of male GCs. CXCL10 activates caspase-8/3 through CXCR3 that in turn induces GC apoptosis (10). Therefore, blocking CXCL10-CXCR3 signaling may alleviate GC degeneration caused by MuV infection.

Laboratory animal models are critical for the studies on the pathogenesis of MuV-induced diseases. Unfortunately, mice are not susceptible to MuV infection. Although MuV efficiently replicates in mouse testicular cells *in vitro*, this is not evident *in vivo* (11). The detrimental effects of ZIKV on the testis are only occurred in mice lacking interferon signaling but not in WT mice (88). A recent study showed that mice lacking type 1 interferon signaling were susceptible to MuV infection (71). Whether orchitis is generated in *Ifnar1* ^−/−^ mice after MuV inoculation remain unknown, and thus requires further investigation. Alternatively, a mouse cell-adapted MuV strain may be used to establish an orchitis model, but how far the observations in mouse models are relevant to human remains questionable.

## Conclusions

The recent outbreaks occurring in highly vaccinated populations have sparked renewed interest in mumps and complications, particularly orchitis. There is a growing concern that a group of mumps cases has shifted from children to young adults and is associated with a high rate of orchitis and severe reproductive problems. The mechanisms behind the development of mumps and orchitis are unknown. Several recent studies on MuV based on primary cells have improved our understanding of mumps virus pathogenesis with regard to MuV receptors-testicular cells interaction, innate immune responses to MuV infection, and detrimental effects on testicular function using mouse models. However, a number of knowledge gaps remain. MuV can effectively replicate in mouse testicular cells *in vitro*. The testis is an immunoprivileged organ for the protection of the spermatozoon from adverse immune response (89). Whether the testicular immune privilege status provides a refuge for MuV replication to escape immune surveillance requires clarification. Rare orchitis cases after the MMR vaccination were reported, suggesting a potential risk of the vaccination (38–41, 71). The pathogenesis of the vaccination-related orchitis remains uncertain and is worth investigating further. In-depth understanding of these questions would help in the development of preventative and therapeutic approaches for mumps orchitis and male infertility.

## Author Contributions

DH and HW designed the concept and wrote the manuscript. DT and FW collected materials and prepared figures. All authors contributed to the article and approved the submitted version.

## Funding

This work was supported by grants from the National Key R&D program of China (Nos. 2018YFC1003900 and 2016YFA0101001), and the National Natural Science Foundation of China (Nos. 81701430 and 82071633).

## Conflict of Interest

The authors declare that the research was conducted in the absence of any commercial or financial relationships that could be construed as a potential conflict of interest.
